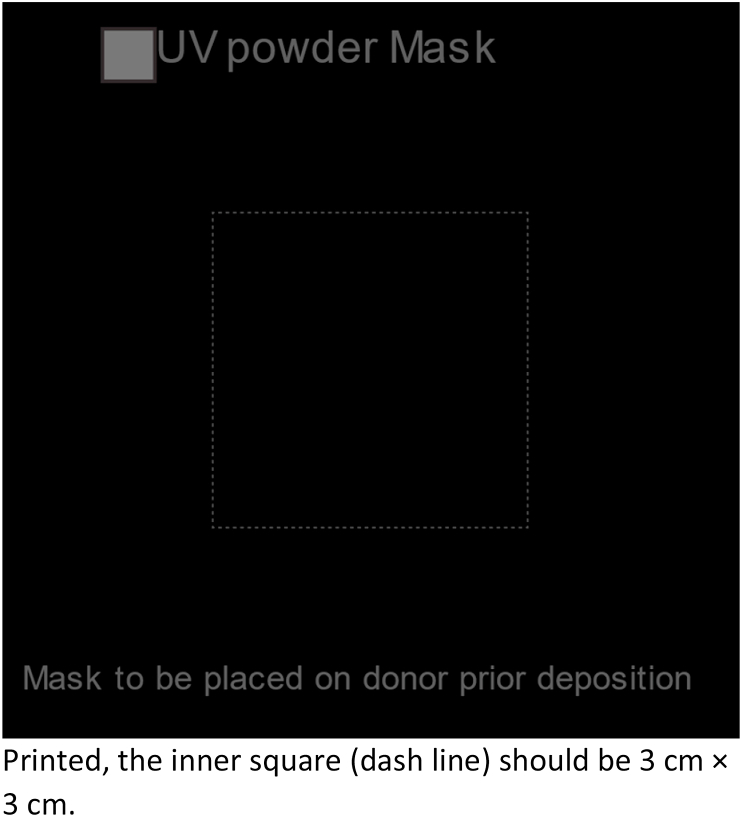# Creation of a universal experimental protocol for the investigation of transfer and persistence of trace evidence: Part 1 - From design to implementation for particulate evidence

**DOI:** 10.1016/j.fsisyn.2021.100165

**Published:** 2021-08-29

**Authors:** Hervé Ménard, Christian Cole, Alexander Gray, Roy Mudie, Joyce K. Klu, Niamh Nic Daéid

**Affiliations:** Leverhulme Research Centre for Forensic Science, University of Dundee, Dundee, DD1 4HN, UK

**Keywords:** Transfer and persistence, Universal protocol, Trace evidence, Open access

## Abstract

Understanding the transfer and persistence of different types of trace evidence between different donor and receiving surfaces under specific conditions, circumstances and alleged competing defence and prosecution hypotheses is a significant need. Acquiring such a knowledge base enables hypothesis testing to be undertaken more readily and with greater confidence. A longstanding goal has been to develop a unified approach to transfer and persistence studies which are fit for purpose but also scalable.

Here we propose a low cost, universal experimental protocol using a recognised and well researched proxy material for the development and aggregation of ground truth transfer and persistence data at scale. We also propose and provide the tools to enable the creation of an open source and open access data repository of experimental data to act as a resource for practitioners and researchers in addressing transfer and persistence questions.

## 1 Introduction

The term ‘trace evidence’ has been used both within the forensic science literature and forensic science and legal practitioner communities to describe a wide range of materials, usually microscopic, most often deposited onto surfaces or transferred between individuals and/or surfaces after a contact or an action has occurred. Typical examples include DNA, body fluids, fingerprint residues, fibres, hair, glass, paint, gunshot residue, explosive or drug residues, ignitable liquids, soil or pollen. The presence of such material, once detected, recognised and compared with a known reference, provides an opportunity to; (i) include or exclude a potential source for the material; (ii) corroborate or otherwise an alleged activity and on some occasions, (iii) provide a potential aid to the identification of a specific material or an individual. Trace materials can thus provide valuable information relating to the determination of source, sub source and activity level propositions [1,2].

Understanding the transfer and persistence of trace evidence is important in addressing, in particular, activity level propositions during the calculation of, for example likelihood ratios, and in the preparation of relevant evaluative opinions. As such, understanding three fundamental and foundational issues are paramount if the scientific findings derived from the analysis of trace materials are to be of most value; (i) how do materials transfer from a source to a receiving substrate? (ii) once transferred, how long does the transferred material persist? and, (iii) what background abundance/prevalence of the transferred material is considered ‘normal’? This work pertains to the first two of these issues.

Material may transfer and persist in different ways where the characteristics of such activities are influenced by parameters such as temperature, humidity, force and duration of contact, and potential activity that gave rise to the contact. One such example is the physical property of fibres being affected by environmental conditions and activities [3,4]. Previous research into the transfer and persistence of trace particulates has most commonly been undertaken either by forensic science practitioners as ad-hoc experiments (often related to forensic case-based questions) or as part of undergraduate, Master's degree, or more occasionally PhD research projects. In particular pollen [[5], [6], [7], [8], [9], [10]], fibres, glass and paint (see for example the review by Trejos et al. [11], the INTERPOL International Forensic Science Managers Symposium report by Almirall et al. [12] and references within) or soil [13] have all been studied by different groups over the last few decades. While much of this work may have generated useful data, most remains unpublished and/or inaccessible. Where reference datasets do exist, for example those listed for hair, fibre, paint and glass in Trejos et al. [11] the collections are mostly maintained within forensic science laboratory facilities, are often created in an ad-hoc way using casework samples or surveys for training and are not publicly available. Even though calls have been made for greater transparency in several landmark reports, National Research Council of the National Academies [14] and the President's Council of Advisors on Science and Technology (PCAST) [15], and again more recently in the latest INTERPOL International Forensic Science Managers Symposium review papers (see for example [16]), the contents of databases remain mainly inaccessible.

It is increasingly recognised that greater open access to data enables scientific development to flourish both within and outside of forensic science, promotes civic participation through citizen science based endeavours and increases collaboration and knowledge-sharing [17,18]. In their recent review, Trejos et al. concluded that forensic science can successfully rise to future challenges with the combined effort of academic researchers, statisticians, laboratory managers and forensic practitioners [11]. This approach is welcomed and sows the seeds for a developmental opportunity for a much wider engagement to harness the enthusiasm and nascent power of the student populations (undergraduate and postgraduate) to truly develop scalable solutions for data gathering.

In 2016, the Leverhulme Research Centre for Forensic Science (LRCFS) was established at the University of Dundee, Scotland. LRCFS has a remit to bring together all the relevant parties from across the law enforcement, legal, judicial and forensic specialisms as well as involving people working outside the criminal justice field to work together with the aims of delivering robust scientifically valid solutions to some of the grand challenges facing the use and implementation of science for the justice systems. This includes exploring solutions for the development of robust datasets which could inform the interpretation and evaluation of trace evidence in multiple scenarios.

A strategic conversation (a workshop which uses a creative design thinking approach to bring whole ecosystems together to create solutions to difficult interdisciplinary challenges) was held to discuss the future of trace evidence. Participants included judges, lawyers, forensic and other scientists, academic scholars, science communicators, citizen scientists and young people from across the Globe. They debated the transfer, persistence and background abundance of trace evidence developing research pathways to address the challenges identified. The concept of a whole ecosystem generated solution is similar to other collaborative scientific endeavours such as the human genome project (HGP) [19] and the discovery of the Higgs boson by the ATLAS and CMS experiments at CERN [20] where multi-institutional groups work together to understand fundamental science. In order to achieve their objectives, common standards in instrumentation, data formats and protocols were required, such as the Ensembl project to store and manage the human DNA data at the Sanger Centre in Cambridge, UK [21].

The scale of the transfer and persistence question in forensic science is of fundamental importance and by spreading the demands across many institutions and groups, a shared endeavour where many small contributions add up to significant data collections can be realised. Such an approach has been successful in particular by engaging with citizen scientists via platforms like Zooniverse (which came from the Sloan Digital Sky Survey needing help to classify galaxies in their data) with hundreds of thousands of volunteers and millions of classifications being made [22,23]. The platform has expanded to include all fields of science and beyond from identifying animals on the Serengeti [24] to the transcription of anti-slavery manuscripts [25] and demonstrates the willingness, enthusiasm and capabilities of citizens to successfully engage in, sometimes complex, scientific tasks and decision making.

The outcomes and conclusions of the LRCFS strategic conversation demonstrated that a series of ‘universal’ transfer and persistence experiments could be developed to produce data that would be valuable and made openly available as a means of creating regio-specific but globally aggregated and curated datasets which would address basic transfer and persistence questions. These outputs were further refined through a collaboration with academics from six UK universities to develop and test a protype universal experimental protocol. By making the experimental data openly available, any interested parties can contribute with the aim that many experimental outputs can be collected from, for example, university undergraduate and postgraduate programs leveraging the activities of their students. The initial template for the universal experiment was created from this collaborative group and uniquely included a whole ecosystem perspective from the crime scene to the courtroom enriched by the knowledge and experience of the academic, science communication and citizen science communities. The ecosystem coming together in this way to solve a common problem provides a powerful impetus for the adoption of a universal experimental approach, engaging volunteers (and collaborators) to perform transfer and persistence experiments and share the data.

The objective of the universal experimental protocol is to create a detailed and specific methodology to generate and, importantly, collect complementary data that will;

(i) test the inter- and intra-variability between participants in relation to the transfer and persistence of materials,
(ii) develop context specific information which can be useful to inform the calculation of likelihood ratios in both source and activity level evaluative opinions and,
(iii) create a baseline of knowledge that enables further research to be undertaken including but not limited to, the creation of algorithmic models for the behaviour of trace materials during contact and subsequent activity on a range of different surfaces.

In order to perform the large-scale comparisons of data collected by disparate groups, it is essential that the same experimental protocol is followed by the participants involved in the work. It is equally critical that the results are reported completely and consistently otherwise the data generated by different groups will be constrained in how they can be compared. Open and transparent data standards are also necessary for the preservation and archiving of data in the long-term [26,27].

In this paper we present the universal experimental protocol for the transfer and persistence of physical trace evidence. We present the proof of concept of the universal experimental protocol in part 2 which provides early data generated by the universal experimental protocol implemented across different universities.

The transfer and persistence protocol for physical trace evidence uses UV powder as a proxy which has been accepted in previous studies [7,9,10,[28], [29], [30]]. In order to achieve the required level of consistency within the generated data, a detailed and prescriptive experimental protocol was required detailing, for example, which material will be used and how the data are captured, named, uploaded to a dataset, curated and accessed. Much of the protocol centres on a "baseline" experiment where several variables are prescribed and controlled. Once researchers or practitioners using this protocol are comfortable with the "baseline" experiment it is envisaged that they will then be able to extend and expand the experiment to move away from the proxy material and follow the protocol to undertake experiments with specific particulate evidence types, donor and receiver materials of more relevance to case specific circumstances. The point being that as a community, a rich dataset can be created on a core set of materials for a range of evidence types, while enabling the addition of information in more niche, yet still important materials, evidence types or conditions. Sharing all the data in a common format to a single unifying open access database means that researchers or practitioners can identify data which they need and/or data which needs further extending or expanding.

The final aspect of the universal experiment is that it is not set in stone. This is version 1.0 of the protocol which has been initially tested by a small group to address initial issues and it is envisioned that it will evolve as demands or needs change. Although the requirements are very specific, we have designed the data capture to be flexible allowing for future improvements and expansions to the protocol. We encourage the forensic science community to get involved with the protocol by adding comments and/or adapting the protocol as needed. For this purpose the protocol is being made openly available via protocols.io, a free online website which makes experiments a (re-)shareable resource which communities can then adapt, adjust and revise.

## 2 Materials and methods

### 2.1 UV powder as a proxy trace evidence

For this protocol UV powder is used as a proxy for trace particulate materials such as dust, drug powders, explosives, sand, gunshot residue, some soils and pollen. UV powder has previously been used in geological studies and while it is accepted that the UV powder will ultimately not make an ideal proxy for all evidence types, it provides a rapid, safe, accessible and low-cost approach to generate reproducible and consistent data in relation to the deposition, transference and persistence of some trace substances. Using a consistent source material allows the collating of all the data as a whole for further analysis. The protocol enables parallel studies to also be performed with material more closely related to evidence types more commonly encountered in casework to determine the reproducibility and repeatability of the experimental process across different sites. Depending on the type of clothing material used, selecting an appropriate UV powder should discriminate between deposited (i.e. UV powder) particles and any background signals.

### 2.2 Equipment

Each experiment provides a means of monitoring the transfer of the UV powder from a donor to a receiving surface using photography and image analysis following a systematised combination of the time vs weight for each transfer event. The following equipment is required and the experiment needs to be undertaken in a dark room or dark cabinet;

• A digital single-lens reflex camera (digital SLR or DSLR) of at least 18 megapixels, which can be mounted and set with a fixed aperture, ISO and shutter speed. A macro setting may be required to get a close-enough picture and the flash needs to be disabled. Optical filters may be required to get the best contrasting results for viewing the transferred powder.
• A humidity sensor and thermometer to record the ambient condition under which the experiment is carried out.
• A UV forensic light source (and appropriate eye protection).
• A set of weights of defined mass (200 g–1000 g, as per Table 1).

**Table 1:** Grid of the baseline experimental conditions for UV powder transfer combinations. The time and mass combinations to undertake the first baseline experiment are shown with a tick mark.

		Contact time (s)			
		30	60	120	240
Mass (g)	200		✓		
	500		✓		
	700		✓		
	1000	✓	✓	✓	✓
• A stopwatch.
• A right angle ruler
• 5 cm × 5 cm swatches of donor (e.g. cotton) and receiver (e.g. nylon, wool, polyester) materials.
• Supportive mounts on which the donor and receiver material swatches are attached prior to the start of the experiment, and a mask card to be used for the UV powder deposition onto the donor sample swatch (templates are available in the Appendix).
• An acrylic (for example Perspex ^TM^) block (3 cm × 3 cm) upon which to place the weight for the transfer step.
• UV powder mixed with white flour (1:3 wt ratio). For the experiments presented in this work, UV powder (green), non-toxic and washable purchased from an online retailer (Fluorescent Neon Ultraviolet UV Blacklight Glow Powder, Green; Zuperpaint; Amazon) was selected.
• Apparatus to achieve a reasonably uniform UV powder deposition on the donor sample.

### 2.3 Experimental setup

**Fig. 1:**
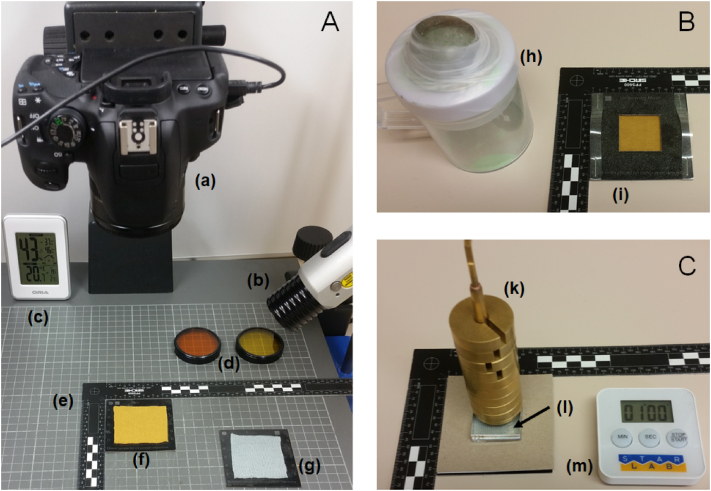
**(A) Setup station for image acquisition:**(a) Camera (connected to a computer (not shown), (b) light source, (c) temperature and humidity sensor, (d) optical filters, (e) Right angle ruler, (f) support mount and donor material, and (g) support mount and receiver material;**(B) Deposition station** (away from other areas), donor sample covered with the UK powder mask ready for UV powder deposition: (h) UV powder, (i) UV powder mask placed on top of the donor material;**(C) Transfer** – the donor sample (on its support mount, underneath and not visible), receiver sample (visible, reverse side of the support mount), acrylic block placed in the 3 cm × 3 cm cutout of the receiver sample support mount and selected mass on top of the acrylic block.: (k) mass resting on top of (l) acrylic block placed in the cut out section of the receiver material) and (m) stopwatch.

Fig. 1 presents an overall view of the equipment required for the experiment to be carried out in a dark room environment. It is recommended the UV powder deposition is undertaken on a different bench and preferably separate room so as to minimise surface contamination. The camera needs to be fixed to a mount at 90° to, and a consistent distance from, the work surface with the rest of the equipment (timer, weights, acrylic block and fabric mounts) within easy reach. In order to avoid variability which may be induced by the movement of the apparatus, the donor material is affixed to a support mount. The support mount is then aligned into the corner of the right-angled ruler placed on the work surface to ensure that the mount does not move during the experiment. When setting up the camera, it is necessary to ensure that the central 3 cm × 3 cm area where the transfer will occur is fully in view. Tethered shooting (connecting a computer to the camera) is recommended to facilitate instantaneous image transfer, reduce the chance of data loss and permit the reviewing of the images on a large screen. We provide a full set of the templates for the support mounts in the appendix.

### 2.4 Camera settings and other equipment set up

**Fig. 2:**
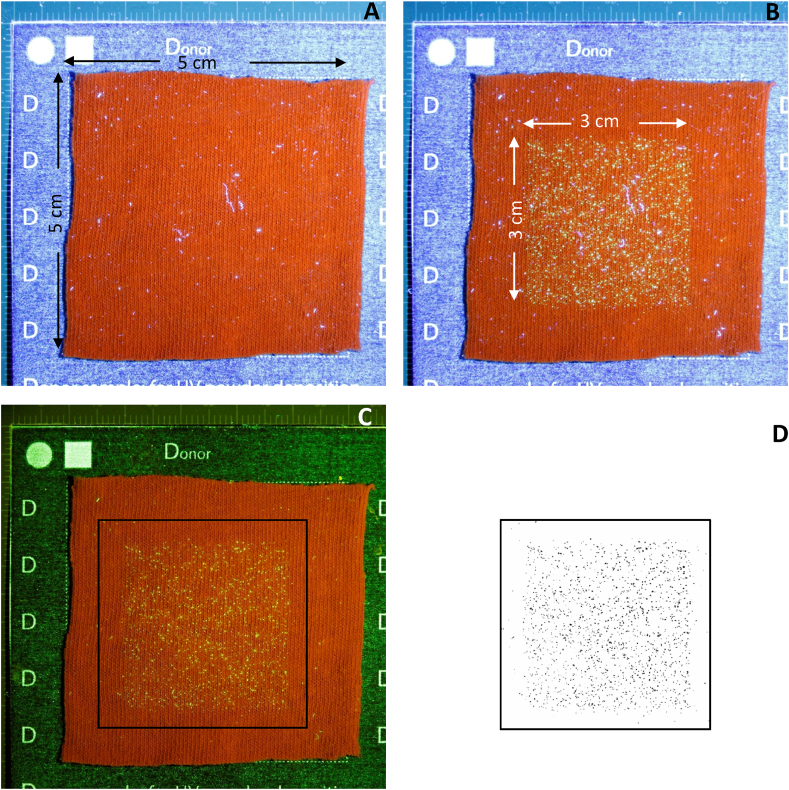
Close up image of the donor material and support mount placed into the corner of the right angled ruler under UV light. (**A)** the donor material prior to UV powder deposition, **(B)** the donor material after UV deposition (large deposition for illustration purposes, the 3 cm × 3 cm deposition area is normally located in the centre of the image), **(C)** the donor material after UV deposition photographed with an additional 510 nm filter on the camera lens and **(D)** after image processing in ImageJ (cropped image marked by the black box in **(C)**, applied threshold value 115, n count 2954 particles). Canon 600D, ISO 400, Aperture 5.6, Shutter speed 4” (exposure condition intended for illustration purposes).

The camera is the primary data source and its operation is critical to obtaining reliable and reproducible results. All images must be taken under the exact same conditions set manually (zoom, shutter speed, ISO and aperture) and saved in the highest quality jpeg mode. The sample must be uniformly illuminated using a UV light source fixed on a mount to ensure consistent lighting throughout the experiment and the camera flash should be disabled. An example image is shown in Fig. 2.

Background particles and fibres are often visible on the surface of the donor and receiver materials once illuminated by the UV light source. These can be distinguished from the deposited and transferred UV powder by either using an appropriate filter on the camera lens (Fig. 2(c)), or as part of the data analysis. Any filter selection must be consistent throughout the data collection and must be reported as part of the metadata files during the data submission.

A very fine deposition of the UV powder/flour mix onto the “donor” material is required. The fine deposition has to be such that the individual grains are observable in the images and no large agglomeration or “clumps” of powder are present. Fig. 2(B) and (C) show an example of the fine deposition required and we found a powder shaker (Fig. 1 (l)) to be a suitable means of achieving this.

### 2.5 Transfer experimental protocol

Two 5 cm × 5 cm swatches of materials are chosen: identified as either the “donor” or “receiver” material. They are mounted on their respective support cards as demonstrated in Fig. 1(f) and (j), using small pieces of Sellotape and without stretching the fabric. The materials must be placed such that the entire central area of the support mount is covered, (marked on the support mount template in the Appendix). The support mount for the receiver material has a 3 cm × 3 cm cut-out in the centre of the mount to allow for the positioning of the acrylic block for the transfer experiment.

Once the selected materials are on their respective support cards; for example, cotton as donor and nylon as receiver, the following experimental protocol should be completed in a dark room or where the experimental set up is placed within a dark cabinet:

1. Place the donor material on its support mount in position aligned with the corner of the right-angled ruler and under the camera.
2. Check that the camera is set up correctly so that it is focused on donor material with the right-angled ruler and the donor material filling the camera field of view.
3. Wearing appropriate eye protection, Illuminate the material with the UV light.
4. Take a “blank” photograph (No. 1) of the donor material.
5. Replace the donor material with the receiving material on its support mount and take a “blank” photograph (No. 2) of the receiver material. Remove the receiver material to one side once the photograph has been taken.
6. Move to a separate ‘deposition station’ away from the camera set up, preferably on a different bench and in a different room. Place the UV powder mask (template available in the Appendix) on top of the donor material and finely deposit a little UV powder onto the surface of the donor material.
7. Carefully remove the UV powder mask and move the donor material back to the camera, position it as before using the right-angle ruler and take a photograph (No. 3) of the deposited UV powder prior to transfer.
8. Perform the transfer between the donor and the receiving materials as follows;
  (i) place the receiver material on top of the donor material such that the two material surfaces are touching each other,
  (ii) place the acrylic block into the 3 cm × 3 cm cut-out area of the support mount of the receiving material and,
  (iii) place a weight of the required mass on top of the acrylic block for the required time period as measured on the stopwatch.
9. After the chosen time has elapsed, carefully separate the donor and the receiver materials and take a photograph (No 4) of the donor material and then (No 5) of the receiver material (by removing the donor material and positioning the receiver material as before under the camera – step 5).
10. Repeat steps 1 to 9 five more times.

**Fig. 3:**
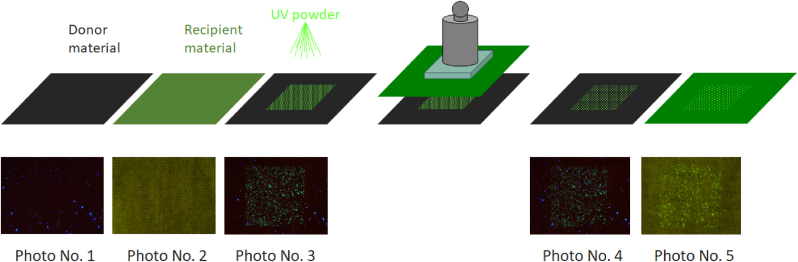
Illustrative steps for the UV powder transfer experimental protocol showing the 5 photographic images (per experimental replicate) taken at each of the different stages.

The capture of each photograph is illustrated diagrammatically in Fig. 3. Each experiment should have 5 photos and the experiment should be replicated 6 times, giving a total data set of 30 photographs for the same combination of donor and receiver material, mass and time of contact.

The contact time and the applied weight for each of the baseline experiments is given (identified by a tick mark) in Table 1. In the protocol the 3 cm × 3 cm deposition area and the maximum contact weight of 1000 g are purposely set to simulate the pressure applied by a 100 kg person sitting on a chair. For the baseline experiments, the donor material should be 100% cotton and either 100% wool or 100% nylon as receiver materials. In each case 5 photographs should be taken per experiment and each experiment repeated 6 times for the 7 time-weight series (Table 1). This will generate a total of 210 photographic images per material combination (e.g. cotton to nylon) for the baseline experiment.

The camera settings will depend on the choice of illumination and filters if used. Multiple series of images collected under various camera settings may be collected to evaluate their effect on the trends. Each of these conditions should be recorded as part of the metadata submission and with 6 replicates for each setting. A comparison of multiple camera setting is discussed in part 2 of this paper series.

In addition to the baseline experiments, extra mass/time combinations can be collected to make the dataset even more comprehensive, for example simulating very short time or light contacts. Other material combinations can be also investigated, such as for example to include denim or polyester.

The fluorescent property of the UV powder makes it ideal for quick and automatic counting of particles through analysing photographic images of the surface using the standard open source software ImageJ, and by so doing, removing the tedious task of manual counting and associated potential increases in measurement error or variability.

### 2.6 Persistence experimental protocol

The persistence experiment is an extension of the transfer protocol with photograph No. 5 of the transfer experiment being the *t*_*0*_ data point in the persistence experiment. These experiments require the ‘wearing’ of the receiver material and as such ethics committee approval may be required.

**Fig. 4:**
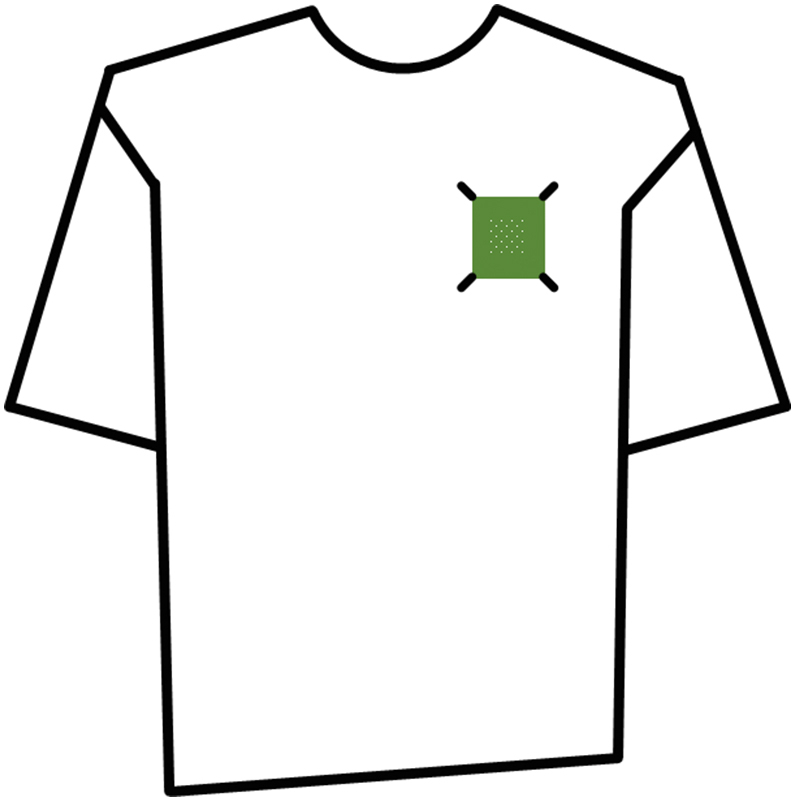
Example of swatch location for the persistence experiment.

The following experimental protocol should be completed;

1. Once the transfer experiment has been undertaken and the photograph of the receiver material captured, the receiver material should be carefully taken off its support mount and attached to a T-shirt or similar garment using four safety pins, one at each corner of the receiver material swatch (Fig. 4).

**Fig. 5:**
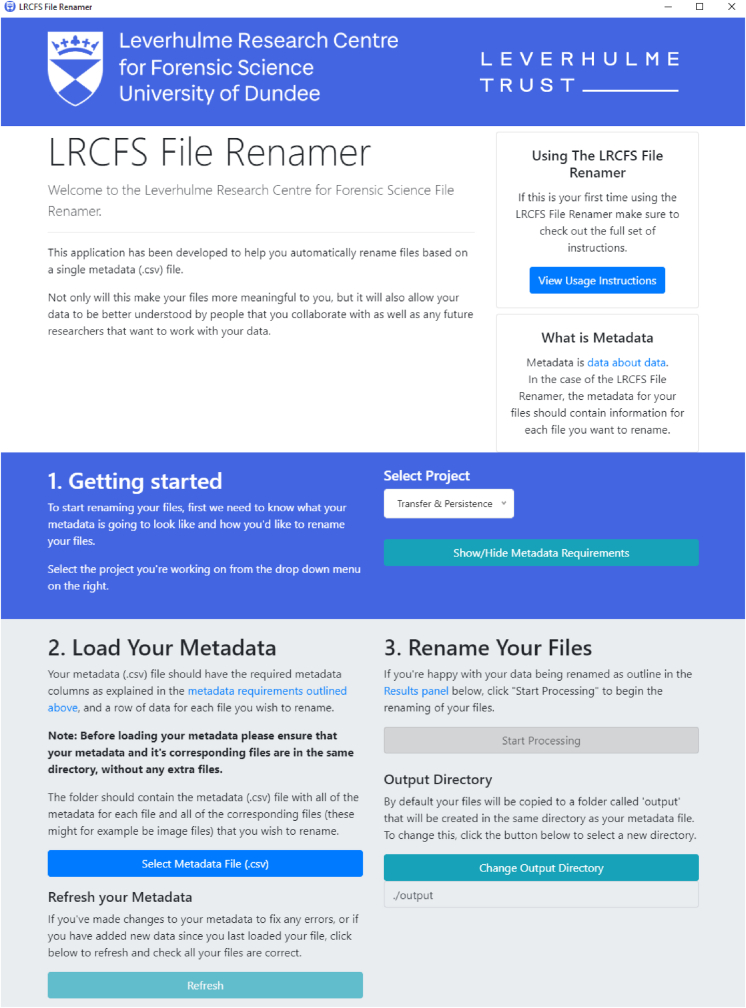
A screenshot of the LRCFS file renamer [31].
2. A baseline experiment should be undertaken as follows;
  (i) The garment with the receiver material swatch attached should be worn by a volunteer/participant. The receiver swatch must be worn uncovered for the entire experiment and the participant should undertake normal activities.
  (ii) Photographic images of the fabric swatch – where the receiver material swatch is removed from the clothing prior to photography and replaced after photography – should be taken at the following times: 30 min, 60 min, 120 min, 180 min, 360 min, 720 min (12 h), 1440 min (24 h), 10080 min (168 h or 1 week), 40320 min (672 h or 4 weeks) using the same camera and lighting set up as per the transfer experiment. The swatch can be attached to different garments for the longer running persistence experiments. The measurements taken after a time greater than 24 h can be considered in days and weeks assuming the fabric swatch was worn for at least 8 h a day.
3. In addition to the baseline experiment;
  (i) Different starting conditions for transfer time/mass and materials could also be undertaken.
  (ii) An extension to the baseline experiment could also be considered where the fabric swatch is attached to the garment in different places (i.e. torso – front, back; leg – upper, lower; arm – upper, lower). Such extensions to the baseline experiment would need to be documented in the metadata prior to submission of the data.

### 2.7 Image analysis

The analysis of the images captured in both the transfer and persistence experiments provide information relating to the number of particles which have transferred from the donor material to the chosen receiver material and the efficiency of that process. The number of particles visible on the acquired photographs can be determined using an appropriate image analysis software package. No specific recommendation is made regarding the selection of a suitable image analysis software package; the participant is free to use one they are familiar with. For the images presented in this paper, ImageJ was chosen because it was open source and facilitated the opening and processing of one or a series of images. This included the ability to undertake a particle count using the same set of parameters and commands recorded in a “macro” using the “command recorder” function which saves, in a text file, every action and click performed by the user. The saved macro can be subsequently run to generate an output for all images saved within the same folder. The particle counting is obtained via the “Analyze Particles” function which applies to ‘thresholded’ or binary images.

The development of the macro is straightforward, an example is provided in Table 2. The code used will be dependent upon the specific conditions under which images are acquired and users can refer to the online manual and other support provided by the user community.

**Table 2:** Example of ImageJ macro written for batch processing images.

makeRectangle(498, 462, 2256, 2178);
run("Crop");
run("8-bit");
setThreshold(20, 255);
setOption("BlackBackground", false);
run("Convert to Mask");
run("Analyze Particles … ", "display clear summarize");

### 2.8 File management

As previously mentioned, the “baseline” transfer experiment constitutes seven combinations of time and mass resulting in 210 raw images for processing and analysis. Commercial cameras do not typically have good file management tools and so will generate filenames such as IMG_0001.jpg, IMG_0002.jpg, etc.

As part of this universal experimental protocol, a tool has been developed together with a structured file format for combining the image file with its associated metadata. Used together, they aim to reduce the presence of typographical errors, missing, incorrect or inconsistent information using a simple, platform-independent interface. The open source tool, called the LRCFS file renamer, was written in javascript using the electron.js framework to ensure that it would work transparently on any platform (e.g. Windows, macOS and Linux), run fast and be configurable. It is available to download from https://doi.org/10.5281/zenodo.4745515.

The absolute minimum metadata required to be captured and collected for every image are as detailed in Table 3. This information is required in order that the data can be aggregated together in a dataset that facilitates the data sharing aspect of the protocol.

**Table 3:** Experiment metadata. For each image the follow items of information need to be collected. The abbreviations are used by the file renamer during image file renaming.

Metadata (units)	Abbreviation	Format
Date	–	YYYYMMDD
Experiment	EX	Number (1-n)
Replicate	RE	Number (1-m) with m≥6
Substrate	SB	String (e.g. cotton)
SubstrateType	ST	‘D’ (donor) or ‘R’ (receiver)
ObservationType	OT	‘Ndata’ (blank) or ‘Sdata’
EvidenceType	ET	String(e.g. none, UVpowder, soil, glass …)
Mass (g)	MA	4-digit number^a^
TransferTime (s)	TT	4-digit number^a^
PersistenceTime (min)	PT	4-digit number^a^
Temperature (°C)	TP	2-digit number^a^^,^^b^
Humidity (%)	HM	3-digit number^a^^,^^b^

a Numbers with fewer than the required number of digits are zero-padded e.g. 0020.

b Fractional values should be rounded to the nearest integer or the nearest even integer at .5 decimal e.g. 21.5 -> 22 as does 22.5 -> 22.

Fig. 5 shows the user interface for the LRCFS file renamer tool. The file renamer converts sequential, camera-derived file names into meaningful and interpretable information e.g. IMG_0001.jpg becomes 20210503_EX1_RP1_SBCotton_STD_OTNdata_ETnone_MA0000_TT0000_PT0000_TP22_HM30.jpg which can be deciphered via Table 2 to mean that the image was generated for Experiment 1, Replicate 1 on the 3^rd^ May 2021 and the material type was a cotton “donor” for a blank with no mass, transfer time or persistence time set. As it is a background image (i.e. “OTNData”, the evidence type is also “none”. The temperature was 22 °C and relative humidity was 30%.

### 2.9 Data sharing

The final step in the universal experimental protocol is to share the results of the experiments with the wider practitioner and research community. A web application was developed to upload the research data generated from experiments undertaken using the protocol which complies with the data format and metadata requirements specified previously. The raw image data and metadata files are automatically parsed and the information stored in a MySQL database and combined with all data contributed by all community participants using this protocol. The LRCFS uploader tool has been developed with the uppy.io open source javascript library which enables fast, efficient and robust uploading of files.

**Fig. 6:**
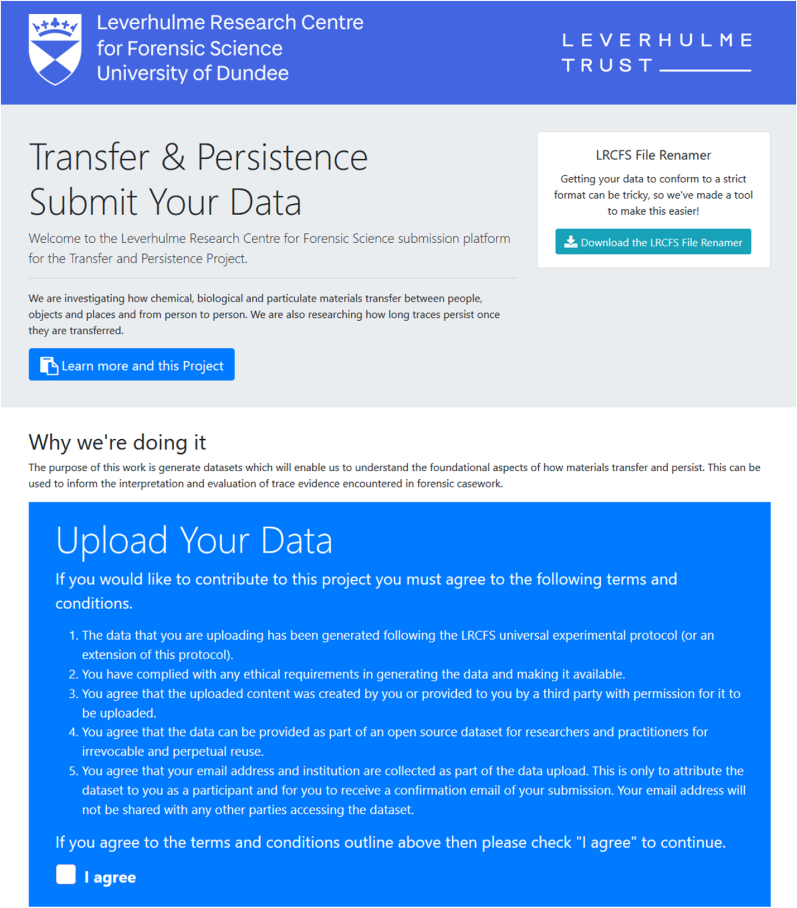
A screenshot of the LRCFS uploader web application [32].

Prior to using the LRCFS uploader, the image files need to be renamed using the LRCFS file renamer and a file list of the metadata created (for example as an excel file). Both the metadata list and all of the image files should then be stored in the same folder on a computer. The LRCFS uploader can be opened via the web link and performs two functions; firstly the image filenames are extracted from the metadata and the uploader verifies that all the files exist and that there are no additional files. Secondly, the metadata and associated images are then uploaded to secure storage on the LRCFS research servers at the University of Dundee ensuring there are no duplicates. If problems are encountered in uploading the files, meaningful error messages are provided directing the user to fix specific problems. Upon successful uploading of data the researcher receives a certificate acknowledging the submission. Fig. 6 shows a screenshot of the LRCFS uploader application. The uploader can be found at https://lrcfs.dundee.ac.uk/transfer-and-persistence-submission/ [32].

## 3 Discussion

### 3.1 Development of the protocol

By creating and detailing a universal experimental protocol the aim was to reduce the variability and improve the reproducibility of experimental data derived from the experiments and to then enable this data to be uploaded to a universal aggregated dataset. The universal experimental protocol as presented here is version 1.0 of an open protocol encouraging the forensic science research community to contribute data and suggest improvements. Prior to this release, the protocol was trialled at three different Universities where challenges in the naming of the generated data led to the development of the LRCFS file renamer tool which turns data files into well-structured project information. Although developed specifically for this experiment, it is of use to any research study requiring the management of large numbers of raw files.

The next challenge was the collation of the raw and processed data and results. In part this was linked to the difficultly of consistently managing hundreds to thousands of files from several experiments. Originally, a fixed format spreadsheet file was disseminated to researchers which they would fill in with the metadata and then return together with the raw image data using a cloud data service such as Box, OneDrive or DropBox. A significant amount of time was required to manually verify and correct inconsistencies in metadata and or filenames. As a result, the LRCFS uploader was developed to create a method for simplifying the verification and uploading of the files.

### 3.2 Using the universal experimental protocol

A core objective of this work was for two outcomes to be supported synergistically;

• to generate the much needed ground truth datasets for transfer and persistence of particulate materials using an appropriate proxy material,
• to create a simple experimental protocol for a low cost, high impact activity which could be incorporated into undergraduate or Masters degree level laboratory practical exercises or as the foundation for research projects. Equally the experiments designed could be undertaken by forensic science practitioners.

The protocol lends itself to both undergraduate and Masters level laboratory based practical exercises in the following ways.

At undergraduate level, the experiments can be run as the baseline experiment only for transfer and persistence. This provides an ‘off the shelf’ practical of variable duration to be run and systematically managed through the variation of the donor and receiving materials being provided to different students within a practical session. A full baseline study including data renaming and uploading should take between 2 and 3 h if all of the materials are in place (4 min per experiment and 12 min to write the metadata file and submit the data). The persistence experiments can be adapted to generate baseline information relating to persistence from the variable starting points of the transfer experiments varying time and weight as well as donor and recipient materials and running the experiments across a time frame that fits with practical sessions. The experiments provide practical exercises in measurement, data interpretation and problem solving all of which would align with requirements of undergraduate practical exercises.

At Masters degree level and for practitioners, the baseline experiments create a means of verifying and validating the experimental setup and the protocol then provides the methodology to use a variety of substances in replacement of the UV powder to investigate specific trace materials such as fibres, glass, soil etc so long as a methodology for their effective visualisation can be developed. This data can also be uploaded to the data set via the file renamer and uploader where the ‘EvidenceType’ field is used to reflect the nature of the sample. The universal experimental protocol enables the development of analytical and interpretative skills at undergraduate and post graduate level through the baseline experiments. Secondly, the baseline experimental results allow for a meta-analysis of groups, researchers and laboratories to determine the consistency and natural variability in the predefined set of experimental parameters presented in the universal experimental protocol including for example external factors such as regional variation in donor and receiver materials or the effects of temperature and humidity.

This project is an example of how the research and practitioner community can come together to develop a protocol to address an important challenge facing forensic science: what does transfer and persistence look like for different materials and trace evidence types? The protocol refers to UV powder as a proxy which acts as a springboard to further studies and development.

## 4 Conclusions and future directions

It is our intention that this is large, long-term project with the potential to generate large amounts of data to answer many open research questions regarding the transfer and persistence of physical, particulate trace evidence material. Using a proxy UV powder is intentional for simplifying the detection aspects of the analysis, however, new experiments can be developed and the protocol extended to include ‘true’ trace evidence types such as for example, GSR, soil or explosives. The approach we have adopted lends itself to the development of modelling approaches of the transfer and persistence phenomena using the UV powder proxy which can be then compared to the behaviour of the ‘true’ evidence types. Differences and similarities can be identified and the probity of any numerical analysis can be assessed. We are throwing out a call to arms across the academic community globally to become involved in this endeavour and to help us develop a large scale database for the benefit of our operational forensic science colleagues. Participation in this project is open to all and interested groups and universities are invited to contact the authors or the Leverhulme Research Centre for Forensic Science (LRC@dundee.ac.uk) for further details on how to get involved.

## CRediT authorship contribution statement

Hervé Ménard, Christian Cole and Roy Mudie: Methodology development, Writing – original draft & editing. Alexander Gray and Joyce K. Klu: Writing – review & editing. Niamh Nic Daéid: Conceptualization, Funding acquisition, Writing – review & editing.

## Funding

This work was funded by the 10.13039/501100000275Leverhulme Trust RC-1015-011.

## Declaration of competing interest

The authors declare that they have no known competing financial interests or personal relationships that could have appeared to influence the work reported in this paper.

## Figures and Tables

**Figure:**
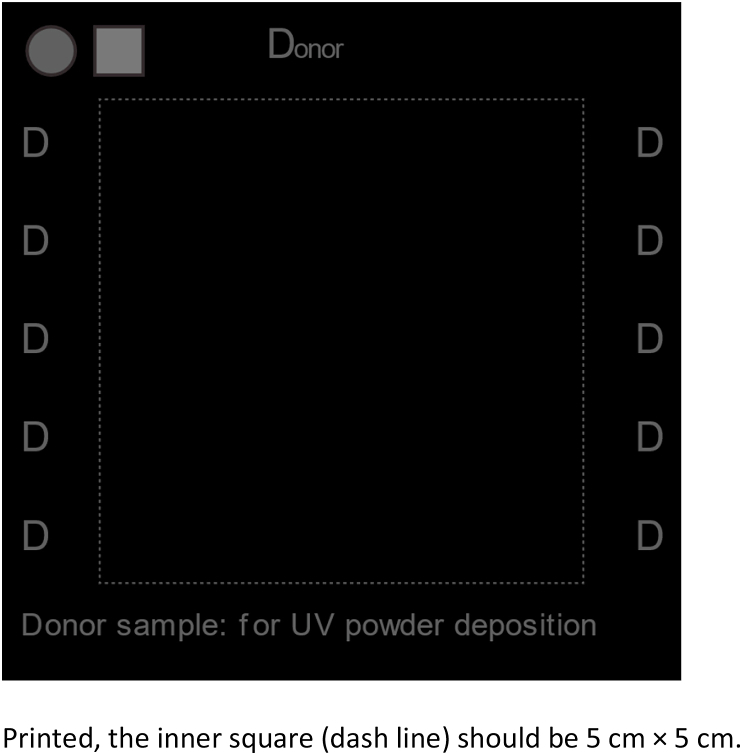


**Figure:**
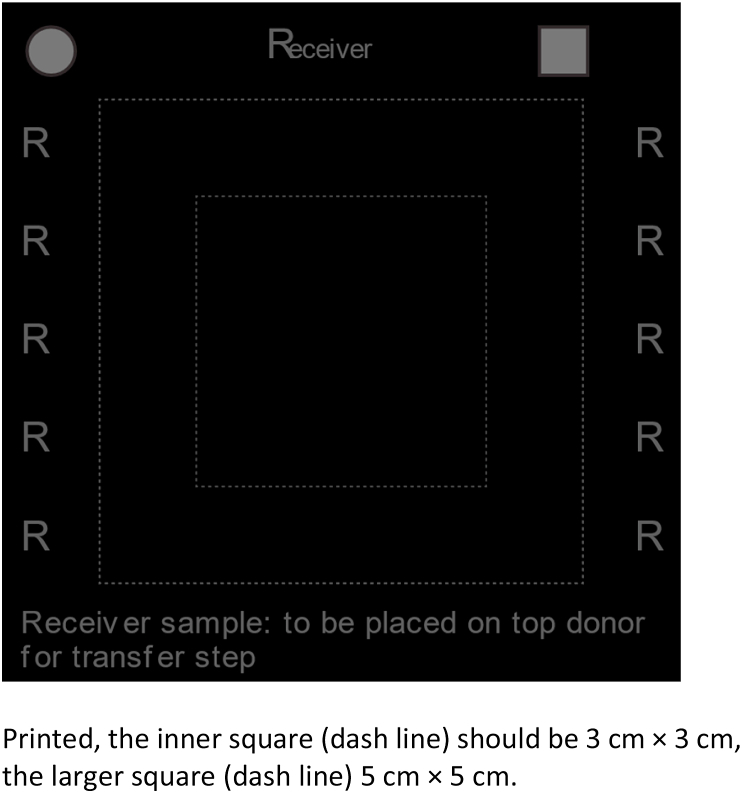


**Figure:**